# Protected Organic Acids Improved Growth Performance, Nutrient Digestibility, and Decreased Gas Emission in Broilers

**DOI:** 10.3390/ani10030416

**Published:** 2020-03-02

**Authors:** Dinh Hai Nguyen, In Ho Kim

**Affiliations:** Department of Animal Resource and Science, Dankook University, Cheonan-si, Chungnam 31116, Korea; nguyenhaibg90@gmail.com

**Keywords:** broiler, growth performance, microflora, nutrient digestibility, organic acids

## Abstract

**Simple Summary:**

Recently, the development of antimicrobial resistance of bacteria has become a global health problem. Such a situation has compelled nutritionists and researchers to explore other potential alternatives. Among a variety of candidates for the replacement of antibiotic growth promoters, organic acids (OAs), both individual and as a blend of several acids are the most promising ones as feed additives in animal production. Organic acids maintain cellular integrity of the gut lining and improve the digestive process by maintaining normal gut flora. Addition of OAs to the diet can improve the absorption rate of proteins, amino acids, and minerals. This may contribute not only in improving performance but also reducing nitrogen and phosphorus excretion. Besides, medium-chain fatty acids (MCFAs) constitute another type of acid and have been shown to be potential alternatives for in-feed antibiotics in farm animals as they have strong antibacterial activity against Gram-positive cocci and *Escherichia coli*. The combination of OAs and MCFAs has been reported to improve the nutrient digestibility, growth performance, proliferation of *Lactobacillus*, and immunity of the animal. The present study investigated the effect of a blend of dietary protected OAs and MCFAs on broiler chickens. The results of this study showed that the blend of OA and MCFA supplementation positively influenced growth performance, DM digestibility, excreta *Lactobacillus* counts, as well as NH_3_ gas emission in broiler chickens.

**Abstract:**

We investigated the effects of a blend of organic acids (OAs) and medium-chain fatty acids (MCFAs) supplementation in 800 1-d-old male Ross 308 broiler chickens (42 ± 0.90 g) in a 7-week study. Broiler chicks were randomly allocated into one of the five dietary treatments (16 birds per pen with 10 pens per treatment). Dietary treatments consisted of corn-soybean meal based basal diet and the basal diet supplemented with blend of OAs and MCFAs at 0.25, 0.5, 0.75 g, and 1 g per kg of feed. In the current study, during the whole experimental period, the inclusion of the blend of OAs and MCFAs in the basal diet linearly improved (*p* < 0.05) body weight gain (BWG), feed conversion ratio (FCR), and dry matter digestibility. The increasing inclusion of the blend of OA and MCFA levels in the diets linearly decreased (*p* = 0.002) feed intake during d 1 to 7. Broilers fed diets containing different levels of the blend of OAs and MCFAs showed a linear increase (*p* = 0.006) in *Lactobacillus* concentrations and decrease (*p* = 0.014) in ammonia (NH_3_) at the end of the experiment. However, the blend of OAs and MCFAs did not affect carcass quality, *E. coli*, and *Salmonella* counts, as well as hydrogen sulfide and total mercaptans gas emission (*p* > 0.05). In conclusion, the blend of OA and MCFA supplementation positively influenced growth performance, DM digestibility, excreta *Lactobacillus* counts, as well as NH_3_ gas emission in broiler chickens.

## 1. Introduction 

Recently, the development of antimicrobial resistance of bacteria has become a global health problem. In such a situation researchers and nutritionists have been compelled to investigate other potential antibiotic alternatives to enhance the performance of poultry [1,2,3]. Among a variety of candidates for the replacement of antibiotic growth promoters, organic acids (OAs), both individually and as a blend of several acids have potential as feed additives in animal production [4]. Organic acids maintain cellular integrity of the gut lining and improve the digestive process by maintaining normal gut flora [5]. Adding OAs to feed can lower gastric pH which accelerates the conversion of pepsinogen to pepsin, thereby improving the absorption rate of proteins, amino acids, and minerals [6]. This may contribute not only to improving performance but also reducing nitrogen and phosphorus excretion with decreasing environmental pollution [7]. OAs have several additional effects that go beyond those of antibiotics. These effects include reduction of digesta pH, increase of pancreatic secretion, trophic effects on the gastrointestinal mucosa [4], and increasing carcass quality such as color of meat, and the firmness of meat or the relative weights of the bursa of Fabricius and spleen. 

Although organic acids have positive effects on growth performance, intestinal microbial growth and health status in animals, the activities of OAs without any protection would be limited due to prompt absorption and metabolism, or both, before undergoing chemical digestion upon entering the duodenum, which eventually inhibits the modulation of intestinal flora [8]. To improve the retention time of nutrients and active compounds in food and drugs, microencapsulation or matrix coating technology was developed, which can slow down the release of those compounds through the gut, in order to enable them to reach the distal part of the intestine in appreciable and effective amounts [9]. This shows that protected organic acids are more effective in delivering OAs to the hindgut of pigs thus improving the growth of beneficial micro-organisms and inhibiting the survival rate of enteric pathogens [10].

Besides, medium-chain fatty acids (MCFAs) constituting another type of acid, have been shown to be good alternatives for in-feed antibiotics in farm animals as they have strong antibacterial activity against Gram-positive cocci [11] and *E. coli* [12]. Recently, it was shown that the supplementation of the MCFA caprylic acid to broiler feed could reduce *Campylobacter* jejuni colonization in these birds, both in a prophylactic way [13] as well as therapeutically [14,15]. With such positive changes the addition of MCFAs may result in better performance of animals, as was found in a previous study [16]. In addition, it has been reported that the combination of an OA and MCFA blend has beneficial and synergistic effects on the performance of piglets by changing the acidity in the upper part of the digestive tract and causing structural changes of the small intestinal mucosa [17,18]. The combination of OAs and MCFAs has been reported to increase the growth performance, proliferation of *Lactobacillus*, and the immunity of weaned pigs [19] as well as the nutrient digestibility in laying hens [20]. However, to the best of our knowledge there is still limited information on the influence of a blend of OAs and MCFAs in poultry. Therefore, the objective of the present study was to assess the effect of a blend of dietary protected OAs and MCFAs on growth performance, nutrient digestibility, carcass quality, excreta microflora, and excreta gas emission in broilers. 

## 2. Materials and Methods

The experimental protocol (DK-1-1729) used in this study was approved by the Animal Care and Use Committee of Dankook University.

### 2.1. Source of the Blend of OAs, and MCFAs

The blend of matrix coated OAs and MCFAs used in the experiment was provided by a commercial company (Morningbio Co., Ltd., Cheonan, South Korea). The active ingredients were 17% fumaric acid, 13% citric acid, 10% malic acid, and 1.2% MCFAs (capric and caprylic acid, provided as a 1:1 mixture product).

### 2.2. Experimental Design, Animals, and Housing 

In the present experiment, a total of 800 one-d-old Ross 308 male broiler chicks with an average initial body weight of 42 ± 0.90 g were used in a 7-week feeding trial. The chicks were randomly allotted into 5 treatments with 10 pens per treatment and 16 birds per pen. Dietary treatments consisted of a corn-soybean meal based basal diet and the basal diet supplemented with a blend of OAs and MCFAs at 0.25, 0.5, 0.75, and 1 g per kg of feed. The composition of the basal diets is shown in Table 1. All diets were formulated to meet or exceed Aviagen’s recommendations (target live weight 1.70–2.40 kg) and were fed in crumble form. [21]. A blend of OAs and MCFAs was included in the diet by replacing the same amount of corn. Broiler chickens were housed in 3 floor battery cages. Room temperature was maintained at 33 ± 1 °C for the first three days and then gradually reduced by 3 °C a week until reaching 24 °C and maintaining for the remainder of the experiment. The relative humidity was around 60%. Broiler chickens received diet and water ad libitum. Each pen had a pan feeder with a 35-cm diameter. Water was provided by evenly spaced nipple drinkers (five nipples per pen) positioned along the side wall of the pen. Artificial light was provided 24 h per day by the use of fluorescent lights. 

### 2.3. Sampling and Measurements

The broilers were weighed by pen and feed intake (FI) was recorded on d 1, 7, 21, and 49 to calculate body weight gain (BWG) and feed conversion ratio (FCR). On day 42, 0.2% chromium oxide (Cr_2_O_3_) (Duksan Pure Chemicals, Asan, South Korea) was added to the diets as an indigestible marker [22] to determine apparent total tract digestibility of dry matter (DM), nitrogen (N), and gross energy. Birds were fed with diets mixed with chromium oxide during days 42–49. On day 49, excreta samples were collected from each pen and stored at −20 °C until analysis. Before chemical analysis, the excreta samples were thawed and dried for 72 h at 60 °C, after which they were finely ground to a size that could pass through a 1-mm screen. All the feed and excreta samples were analyzed for DM (method 930.15, AOAC International, 2007) and N (method 990.03, AOAC International, 2007). Chromium was analyzed using UV absorption spectrophotometry (UV-1201, Shimadzu, Kyoto, Japan). Nitrogen was determined by using an N analyzer (Kjeltec 2300 Nitrogen Analyzer; Foss Tecator AB, Hoeganaes, Sweden). The gross energy was determined by measuring the heat of combustion using an oxygen bomb calorimeter (Parr 6100 instrument Co., Moline, IL). The apparent total tract digestibility was calculated using the following formula: Digestibility (%) = {1 - [(Nf × Cd)/(Nd × Cf)]} × 100, where Nf = nutrient concentration in excreta (% DM), Nd = nutrient concentration in diet (% DM), Cd = chromium concentration in diet (% DM), and Cf = chromium concentration in excreta (% DM).

At the end of the experiment, 20 birds were randomly selected from each treatment (2 birds per pen). They were weighed individually and euthanized by cervical dislocation. The gizzard, breast meat, bursa of Fabricius, liver, spleen, and abdominal fat were removed by trained personnel and weighed. Organ weight was expressed as a percentage of live body weight. The carcass quality was evaluated by measuring the lightness (L*), redness (a*), and yellowness (b*) values of breast meat and were determined using a Minolta CR410 chromameter (Konica Minolta Sensing Inc., Osaka, Japan). Duplicate pH values for each sample (breast meat) were measured using a pH meter (Fisher Scientific, Pittsburgh, PA). The water holding capacity (WHC) was measured in accordance with the methods described by Kauffman et al. [23]. The ratio of water to meat area was then calculated, giving a measure of WHC (a smaller ratio indicates a higher WHC). Drip loss percentage was determined on d 1, 3, 5, and 7 post-slaughter by using the plastic bag method [24]. Cooking loss was determined using 5 g of breast meat, which was heat-treated in plastic bags separately in a water bath (100 °C) for five min. Samples were cooled at room temperature. Cooking loss was calculated as (sample weight before cooking – sample weight after cooking)/sample weight before cooking × 100 [25].

On day 49, one-gram of fresh excreta samples was collected from cloacae into micro-tubes from each pen (10 samples per treatment) directly for the determination of *E. coli*, *Lactobacillus,* and *Salmonella* counts. Excreta samples were diluted with 9 ml of 1% peptone broth (Becton, Dickinson, Franklin Lakes, NJ) and then mixed with a circulator (Seward Stomacher 400 circulator, West Sussex, UK). Ten-Fold serial dilutions of samples were plated onto agar plates in triplicates. The MacConkey agar plates (Difco Laboratories, Detroit, MI), lactobacilli medium III agar plates (Medium 638, DSMZ, Braunschweig, Germany), and *Salmonella shigella* agar plates (Difco Laboratories) were used to isolate the *E. coli, Lactobacillus,* and *Salmonella,* respectively. The *lactobacilli* medium III agar plates were then incubated for 48 h at 37 °C under anaerobic conditions. The MacConkey and *Salmonella* shigella agar plates were incubated for 24 h at 37 °C. The microflora colonies were counted immediately after removal from the incubator. Concentration of microflora was finally expressed as log_10_ colony forming units per gram of excreta.

The noxious emission was determined according to the method described by Cho et al [26]. Briefly, around 300 g of excreta was stored in 2.6 L sealed plastic boxes in duplicates. These samples were permitted to ferment at 32 °C for 30 h. After the fermentation period, an instrument (Gas Detector, GV-100S; Gastec Corp., Kanagawa, Japan) was used for gas detection. The plastic boxes were punctured and headspace air was sampled approximately 2.0 cm above the samples at a rate of 100 mL/min. Levels of ammonia, hydrogen sulfide, and total mercaptans were measured using Gastec Detector Tube No. 3La, No. 4LK, and No.70 L (Gastec Corp.), respectively.

### 2.4. Statistical Analysis

All data were analyzed as a completely randomized design using mixed procedures of SAS (SAS Institute 2004) [27] to determine linear and quadratic effects. Polynomial regression was used to describe the shape of the response to increasing concentrations of protected organic acids in the diets, with the cage serving as the experimental unit. The initial BW was used as a covariate for the BWG. Variability in the data was expressed as standard error. A probability level of less than 0.05 was considered as statistically significant.

## 3. Results

### 3.1. Growth Performance

The data presented in Table 2 show the results of growth performance. Increasing inclusion of the blend of OAs and MCFAs levels in the diets linearly improved the BWG and FCR during d 1 to 7, d 21 to 49, and during the whole experimental period (*p* < 0.0005). In addition, there was a linear decrease (*p* = 0.002) in FI during d 1 to 7 with increasing levels of the blend of OAs and MCFAs in the diets. However, no significant differences were observed in BWG, FI, and FCR during d 7 to 21 as well as FI during d 21 to 49 and during the overall experimental period among five treatments (*p* > 0.05).

### 3.2. Nutrient Digestibility 

The results of nutrient digestibility are summarized in Table 3. There was a linear improvement (*p* = 0.004) in DM digestibility on day 49 with increasing levels of the blend of OAs and MCFAs in the diets. However, no influence of the blend of OA and MCFA supplementation was found in the digestibility of N and retention of gross energy (*p* > 0.05).

### 3.3. Carcass Quality

The data presented in Table 4 show that increasing inclusion of the blend of OAs and MCFAs did not affect pH value, breast muscle color, WHC, cooking loss, drip loss, beast meat yield, and relative organ weight (*p* > 0.05)

### 3.4. Excreta Microflora

Table 5 shows the results on excreta microflora. These results showed that a linear improvement was observed in *Lactobacillus* concentrations on d 49 (*p* = 0.006). Besides, no influence of the blend of OA and MCFA supplementation was found on the count of *E. coli* and *Salmonella* among five treatments (*p* > 0.05).

### 3.5. Excreta Gas Emission 

There was a linear reduction (*p* = 0.014) in the NH_3_ gas emission (Table 6) associated with increasing the levels of the blend of OAs and MCFAs in the diets. No significant difference was observed in H_2_S and R.SH on d 49 on increasing the blend of OA and MCFA levels in the diets among five treatments (*p* > 0.05).

## 4. Discussion

### 4.1. Growth Performance 

Protected organic acid used in the current study is produced through a matrix coating technology. Active ingredients such as citric acid, malic acid, fumaric acid, and MCFAs are dispersed in a matrix of shell material, a lipid which can allow the active components to reach the intestine in an intact form, and be released slowly by the reaction of lipase from the intestine thereby showing beneficial effects to animals [10,28]. It has been previously reported that using the same product of protected blend of OA and MCFA supplementation in diets improved growth performance of piglets, and growing and finishing pigs [7,10,29] as well as laying hens [20]. In this study, we also observed that the BWG and FCR were linearly improved when broilers were fed the diets supplemented with the blend of OAs and MCFAs. The improvements of growth performance are in line with the results of Adil et al. and Sultan et al. who reported that broilers fed the diets with different levels of organic acids such as with citric acid and lactic acid supplementation linearly improved growth performance and FCR [30,31]. Various researchers also reported that the supplementation of organic acids to the diet of broilers chickens had beneficial effects on BWG [32,33] and FCR [34,35]. The reason for improvement might be the direct antimicrobial effect of OAs and MCFAs, which might have resulted in the inhibition of intestinal bacteria leading to the reduced bacterial competition with the host for available nutrients and a reduction in the levels of toxic bacterial metabolites as a result of lessened bacterial fermentation resulting in the improvement of protein and energy digestibility [35]. Furthermore, Odle has shown that MCFAs could be a rapidly available energy source for young animals, due to their direct transport via portal blood to the liver, which may also explain the observed improvement with the blend of OA and MCFA supplementation [36]. The improvement in FCR could possibly be due to better utilization of nutrients resulting in increased body weight gain in the birds fed the blend of OAs and MCFAs in the diets.

### 4.2. Nutrient Digestibility 

Feed additives, such as OAs in the diets are known to support mechanisms for stimulation of intestinal mucosal growth such a reducing growth rate of many pathogenic intestinal bacteria, decreasing the intestinal colonization and infectious processes, and promoting and maintaining an optimal microbiota, which in turn can reduce the presence of toxins that can negatively affect intestinal morphology [37], compromise intestinal integrity [38], and increase the digestion and absorption of nutrients by the mucosa [39]. In the current study we also observed that increasing inclusion of the blend of OAs and MCFAs in the diets linearly increased the digestibility of DM, which is in agreement with Pirgozliev et al., who reported that broilers fed OAs had higher metabolizability coefficients of DM values than those fed diets without OAs [40]. Feeding piglets with the same product (the blend of OAs and MCFAs) also were reported to show increasing DM digestibility [41]. However, the N and energy digestibility in this study were not improved by the blend of OA and MCFA supplementation. In contrast, other studies reported that the DM digestibility was not affected with dietary supplementation of the same product in laying hens [20], organic acids such as formic, fumaric, or lactic acid in pigs [42,43]. Moreover, it was found that the metabolizable coefficients of gross energy and N had increased in broilers fed OAs [40,44] compared to those fed diets without OAs. The different results in the aforementioned studies could be due to the different animal, composition of the diet, and the different amount and type of OAs and MCFAs supplemented.

### 4.3. Excreta Microbial

Gut health is one of the major factors governing the performance of birds and thus, the economics of poultry production [45] while the profile of intestinal microflora play an important role in gut health. Dietary OAs and their salts are able to impair microbial growth in the food and consequently to preserve the microbial balance in the gastrointestinal tract. In this study, we also observed an increase in the concentration of *Lactobacillus* in broilers fed the diet supplemented with the blend of OAs and MCFAs, which is consistent with the result of Gheisari et al., who reported that ileum contents of the birds fed a diet containing 0.2% OAs mixture had significantly higher counts of lactobacilli [46]. In addition, Lee et al. reported that a microencapsulated OAs blend with MCFA supplementation led to increase excreta *Lactobacillus* content of laying hens at 30 and 35 weeks of age [20]. The result of our study was supported by Chowdhury et al., who reported that the use of citric acid produces an acidic environment in the gut thus favoring the development of lactobacilli [47]. The increase of *Lactobacillus* population has positive effects on intestinal function by reducing the survival rate of enteric pathogens due to the low pH resulting from the acid produced by the fermentation of *Lactobacillus*. As the microbial pathogenic population becomes reduced, the metabolic requirements of the microbes is reduced and the availability of dietary energy and nutrients to the host animal is increased, leading to enhanced growth rate and feed efficiency [10].

### 4.4. Carcass Quality

The result of the present study showed that the inclusion of a blend of OAs and MCFAs had no effect on carcass quality in broilers. Similarly, Thirumeignanam et al. found no effect on the carcass characteristics of broiler chickens fed organic acid-based diets [48]. However, another study reported that supplementation of the same product increased the color and the firmness of meat in finishing pigs [29]. Furthermore, Mohamed et al. also reported that addition of protected OAs (mixture of fumaric acid, calcium formate, calcium propionate, potassium sorbate, and hydrogenated vegetable oil) increased the relative weight of the bursa of Fabricius and spleen in broilers [49]. According to D’Alessandro and Zolla, meat quality is affected by lots of factors, including breed, nutrition, husbandry conditions, and handling before and after slaughter [50]. Therefore, we assume that the possible reason of the variation in results could be due to the different animal, inclusion levels and type of OAs, and composition of diet.

### 4.5. Gas Emission 

The emission of odorous gases such as NH_3_, H_2_S, and total mercaptans, are major aerial pollutants originating from animal production [51]. Therefore, to ensure sustainable broiler production, the emission of such odorous gases should be reduced by proper management and dietary modification. In this study, we found that supplementing the diets with 0 to 0.10% of the blend of OAs and MCFAs linearly decreased excreta NH_3_ gas emission in broilers. However, to the bestof our knowledge, there is a scarcity of information on the effects of the blend of OA and MCFA supplementation on excreta noxious gas emission in poultry. Therefore, no comparisons could be made with other studies. In pigs, it has been reported that using the same product (blend of OAs and MCFAs) decreased the NH_3_ gas emission [8,29]. The reduction in NH_3_ gas emission could possibly be due to a decline in the pathogenic bacterial population in the gastrointestinal tract or due to enhancement of beneficial microbial activity, leading to changes in end products of microbial fermentation and a shift in the ecosystem towards being more ecofriendly [29]. In addition, it has been suggested by Yan et al that fecal noxious gas emission is associated with nutrient digestibility because the higher digestibility may result in lower substrate for the microbial fermentation in the large intestine, which consequently decreases fecal noxious gas emission [52]. Therefore, the reduced excreta NH_3_ emission in this study was probably due to the enhanced nutrient digestibility and population of *Lactobacillus.*

## 5. Conclusions

In conclusion, dietary supplementation of the protected blend of OAs and MCFAs improved growth performance and nutrient digestibility, shifted microbiota by raising excreta *Lactobacillus* counts, and decreased excreta NH_3_ gas emission. However, no significant difference was observed in other parameters of carcass quality.

## Figures and Tables

**Table 1:** Basal diet composition of broilers (as-fed basis) ^1^.

Ingredient, %	Starter	Grower	Finisher
Corn	43.410	55.090	58.590
SBM, (CP 45%)	25.70	22.60	19.61
Wheat Bran	10.300	0.300	0.300
Wheat Flour	5.00	5.00	5.00
RSM (CP 38%)	-	2.00	-
Canola	-	2.00	-
Corn gluten	2.90	-	-
Sesame Meal	2.00	2.00	2.00
DDGS (Corn)	3.00	3.00	5.00
Meat meal (CP 60%, Low P)	2.00	3.00	3.00
Tallow	1.00	1.80	3.10
Soy oil	0.50	-	-
Limestone	1.33	1.25	1.29
MDCP	0.77	0.19	0.35
Salt	0.33	0.26	0.24
Methionine (99%, DL-Form)	0.36	0.33	0.34
Lysine (50%)	0.83	0.63	0.67
Threonine (98.5%)	0.19	0.18	0.14
Choline (50%)	0.13	0.10	0.10
Vitamin premix^2^	0.10	0.10	0.10
Mineral premix^3^	0.10	0.10	0.10
Phytase	0.05	0.07	0.07
Calculated composition, %			
Crude protein	21.99	20.49	18.49
Crude fat	4.08	4.95	6.08
Crude fiber	2.44	2.66	2.40
Crude ash	5.85	5.27	5.06
ME (kcal/kg)	3045	3135	3251
Calcium	0.96	0.90	0.89
Total Phosphate	0.57	0.50	0.49
Methionine + Cystine	1.12	0.99	0.93

^1^ Provided starter diets from d 0 to 7, grower from d 8 to 21 and finisher diets from d 22 to 49. Replaced the same amount of corn with the blend of organic acids and medium-chain fatty acids to create dietary treatments; ^2^ Provided per kilogram of diet: 15,000 IU vitamin A; 3750 IU vitamin D3; 37.5 IU vitamin E; 2.55 mg vitamin K3; 3 mg vitamin B1; 7.5 mg riboflavin; 4.5 mg vitamin B6; 24 mg vitamin B12; 51 mg niacin; 1.5 mg folic acid; 126 mg biotin; and 13.5 mg pantothenic acid; ^3^ Provided per kilogram of diet: 37.5 mg Zn (as ZnSO4); 37.5 mg Mn (as MnO2); 37.5 mg Fe (as FeSO4•7H2O); 3.75 mg Cu (as CuSO4•5H2O); 0.83 mg I (as KI); and 0.23 mg Se (as Na2SeO3•5H2O). Abbreviations: CP, crude protein; DDGS, distiller’s dried grains with solubles; MDCP, mono dicalcium phosphate; ME, metabolizable energy; SBM, soy bean meal; RSM, rapeseed meal.

**Table 2:** Effect of coating the blend of OAs and MCFAs supplementation on growth performance in broilers.

Items	CON	TRT1	TRT2	TRT3	TRT4	SEM	*p*-Value		
							Linear	Quadratic	
	d 1-7								
BWG, g	107	111	111	121	119	1.162	<.0001		0.6812
FI, g	152	136	147	135	134	3.501	0.002		0.571
FCR	1.423	1.235	1.318	1.114	1.123	0.034	<.0001		0.405
	d 7-21								
BWG, g	643	645	654	650	680	13	0.058		0.404
FI, g	965	969	968	979	974	62	0.168		0.662
FCR	1.504	1.511	1.481	1.513	1.436	0.028	0.145		0.325
	d 21-49								
BWG, g	2504	2527	2542	2643	2658	47	0.007		0.695
FI, g	5018	5037	5020	5045	5044	44	0.676		0.981
FCR	2.008	1.999	1.977	1.913	1.900	0.027	0.001		0.643
	Overall								
BWG, g	3253	3282	3307	3414	3457	43	0.0003		0.497
FI, g	6135	6142	6134	6159	6152	46	0.739		0.975
FCR	1.887	1.874	1.855	1.806	1.781	0.017	<.0001		0.405

Abbreviation: CON: Basal diet, TRT1: CON + coated organic acid 0.025%, TRT2: CON + coated organic acid 0.050%, TRT3: CON + coated organic acid 0.075%, TRT4: CON + coated organic acid 0.100%, SEM: standard error of means, BWG: body weight gain, FI: feed intake, FCR: feed conversion ratio.

**Table 3:** Effect of coating the blend of OA and MCFA supplementation on nutrient digestibility in broilers.

Items, %	CON	TRT1	TRT2	TRT3	TRT4	SEM	*p*-Value	
							Linear	Quadratic
Dry matter	71.44	72.78	73.26	74.03	74.26	0.68	0.0036	0.4583
Nitrogen	69.12	69.32	69.99	70.31	70.35	0.66	0.1126	0.7842
Energy	71.28	71.31	71.38	71.34	71.51	0.68	0.8120	0.9448

Abbreviation: CON: Basal diet, TRT1: CON + coated organic acid 0.025%, TRT2: CON + coated organic acid 0.050%, TRT3: CON + coated organic acid 0.075%, TRT4: CON + coated organic acid 0.100%, SEM: standard error of means.

**Table 4:** Effect of coating the blend of OA and MCFA supplementation on meat quality in broilers.

Items	CON	TRT1	TRT2	TRT3	TRT4	SEM	*p*-Value	
							Linear	Quadratic
pH value	5.52	5.48	5.49	5.47	5.46	0.03	0.2527	0.7936
Breast muscle color								
Lightness (L*)	52.06	52.14	52.66	53.37	53.00	0.84	0.2517	0.8280
Redness (a*)	10.51	10.86	11.06	10.82	11.06	0.37	0.3661	0.6318
Yellowness (b*)	10.48	10.73	10.72	10.77	10.82	0.37	0.5413	0.8113
WHC, %	52.59	52.78	52.80	52.85	52.96	1.71	0.8824	0.9858
Cooking loss, %	18.31	18.25	18.18	18.20	18.13	0.85	0.8809	0.9811
Drip loss, %								
d 1	2.96	2.60	2.55	2.64	2.45	0.22	0.1676	0.5680
d 3	4.77	4.71	4.70	4.63	4.61	0.22	0.5607	0.9805
d 5	6.24	6.19	6.20	6.17	6.16	0.21	0.7788	0.9609
d 7	9.34	9.30	9.23	9.19	9.21	0.19	0.5448	0.8152
Relative live body weight, %								
Breast muscle	19.28	19.31	19.36	19.37	19.41	0.37	0.7751	0.9867
Liver	2.73	2.77	2.75	2.79	2.85	0.10	0.4165	0.7899
Bursa of Fabricius	0.14	0.14	0.14	0.13	0.13	0.01	0.8701	0.8900
Abdominal fat	1.89	1.84	1.79	1.81	1.76	0.07	0.1825	0.8487
Spleen	0.17	0.18	0.17	0.17	0.17	0.01	0.9406	0.7528
Gizzard	1.59	1.52	1.48	1.48	1.44	0.08	0.1825	0.7523

Abbreviation: CON: Basal diet, TRT1: CON + coated organic acid 0.025%, TRT2: CON + coated organic acid 0.050%, TRT3: CON + coated organic acid 0.075%, TRT4: CON + coated organic acid 0.100%, SEM: standard error of means, WHC: water holding capacity.

**Table 5:** Effect of coating the blend of OA and MCFA supplementation on excreta microflora in broilers.

Items, log_10_cfu/g	CON	TRT1	TRT2	TRT3	TRT4	SEM	*p*-Value	
							Linear	Quadratic
*Lactobacillus*	7.30	7.35	7.37	7.39	7.40	0.03	0.0064	0.3364
*E. coil*	5.98	5.97	5.96	5.97	5.95	0.53	0.7415	0.5642
*Salmonella*	2.85	2.82	2.83	2.79	2.76	0.09	0.4221	0.8985

Abbreviation: CON: Basal diet, TRT1: CON + coated organic acid 0.025%, TRT2: CON + coated organic acid 0.050%, TRT3: CON + coated organic acid 0.075%, TRT4: CON + coated organic acid 0.100%, SEM: standard error of means.

**Table 6:** Effect of coating the blend of OA and MCFA supplementation on excreta gas emission in broilers.

Items, ppm	CON	TRT1	TRT2	TRT3	TRT4	SEM	*p*-Value	
							Linear	Quadratic
NH_3_	19.1	18.8	18.4	17.9	17.2	0.6	0.0136	0.6179
H_2_S	2.0	1.8	1.9	1.8	1.7	0.2	0.1742	0.9454
Total mercaptans	1.3	1.2	1.1	1.2	1.0	0.1	0.1226	0.9519

Abbreviation: CON: Basal diet, TRT1: CON + coated organic acid 0.025%, TRT2: CON + coated organic acid 0.050%, TRT3: CON + coated organic acid 0.075%, TRT4: CON + coated organic acid 0.100%, SEM: standard error of means, NH_3_: ammonia, H_2_S: hydrogen sulfide.
